# AI-Driven Decimeter-Level Indoor Localization Using Single-Link Wi-Fi: Adaptive Clustering and Probabilistic Multipath Mitigation

**DOI:** 10.3390/s26020642

**Published:** 2026-01-18

**Authors:** Li-Ping Tian, Chih-Min Yu, Li-Chun Wang, Zhizhang (David) Chen

**Affiliations:** 1 Fujian Chuanzheng Communications College, Fuzhou 350007, China; 2 Department of Information and Computer Engineering, Chung Yuan Christian University, Taoyuan 320314, Taiwan; 3 Department of Electrical and Computer Engineering, National Yang Ming Chiao Tung University, Hsinchu 300093, Taiwan; 4 School of Physics and Information Engineering, Fuzhou University, Fuzhou 350007, China

**Keywords:** indoor localization, Wi-Fi sensing, channel state information (CSI), unsupervised learning, adaptive clustering

## Abstract

**Highlights:**

**What are the main findings?**

- An AI-driven single-link Wi-Fi CSI localization framework is proposed, achieving decimeter-level indoor positioning accuracy without relying on historical trajectories or multi-link infrastructure.
- A two-stage TOF estimation combined with adaptive spatio-temporal AOA clustering effectively suppresses multipath interference and eliminates cumulative localization errors in NLOS environments.

**What are the implications of the main findings?**

- The proposed approach enables low-cost, real-time indoor localization using commodity Wi-Fi hardware, making it suitable for large-scale deployment in smart buildings and human tracking applications.
- The single-link, training-free architecture provides a robust alternative to fingerprinting and multi-link systems, offering improved adaptability to dynamic and complex indoor environments.

**Abstract:**

This paper presents an Artificial Intelligence (AI)-driven framework for high-precision indoor localization using single-link Wi-Fi channel state information (CSI), targeting real-time deployment in complex multipath environments. To overcome challenges such as signal distortion and environmental dynamics, the proposed system integrates adaptive and unsupervised intelligence modules into the localization pipeline. A refined two-stage time-of-flight (TOF) estimation method is introduced, combining a minimum-norm algorithm with a probability-weighted refinement mechanism that improves ranging accuracy under non-line-of-sight (NLOS) conditions. Simultaneously, an adaptive parameter-tuned DBSCAN algorithm is applied to angle-of-arrival (AOA) sequences, enabling unsupervised spatio-temporal clustering for stable direction estimation without requiring prior labels or environmental calibration. These AI-enabled components allow the system to dynamically suppress multipath interference, eliminate positioning ambiguity, and maintain robustness across diverse indoor layouts. Comprehensive experiments conducted on the Widar2.0 dataset demonstrate that the proposed method achieves decimeter-level accuracy with an average localization error of 0.63 m, outperforming existing methods such as “Widar2.0” and “Dynamic-MUSIC” in both accuracy and efficiency. This intelligent and lightweight architecture is fully compatible with commodity Wi-Fi hardware and offers significant potential for real-time human tracking, smart building navigation, and other location-aware AI applications.

## 1. Introduction

Position technology has become a cornerstone of modern life, profoundly shaping how individuals navigate and interact with the world. In outdoor environments, systems like the global positioning system (GPS) and the BeiDou Satellite Navigation System have long provided reliable and effective solutions. These technologies are widely adopted and have proven effective in diverse applications, ranging from personal navigation to industrial logistics. However, their utility is significantly constrained in indoor settings, where electromagnetic and radio signals are often attenuated or obstructed by architectural features such as walls and buildings. This limitation underscores the pressing need for reliable indoor positioning technologies to address these challenges. The development of such systems has become critical for increasing the demand for precise and dependable indoor localization, with applications spanning from smart home automation to industrial process monitoring.

An effective indoor positioning system holds immense potential across various domains. For instance, it can be used for detecting falls among the elderly, monitoring abnormal behaviors of prisoners, facilitating emergency management [1], optimizing smart energy systems [2], enhancing HVAC controls [3], improving occupancy detection [4], and managing the storage and tracking of valuable goods. To meet these needs, various indoor positioning technologies have been developed, including Bluetooth, ultrasound, UWB radar, infrared, RFID, ZigBee, and light (or video)-based systems. Despite their advancements, these technologies face notable limitations. For instance, Bluetooth operates effectively only within short ranges of approximately ten meters [5,6]. Meanwhile, Bluetooth Low Energy (BLE) is more energy-efficient than Wi-Fi [7], and its localization accuracy is limited to 1–5 m. Ultrasonic-based techniques [8,9] are significantly affected by multipath effects caused by indoor obstacles, which reduced their accuracy. Although UWB-based methods offer higher precision [10,11], they require expensive and complex equipment, limiting their scalability. Similarly, RFID-based systems [12,13,14] are susceptible to interference, and light- or video-based localization techniques [15,16] raise privacy concerns and rely on consistent lighting conditions. Given the widespread availability of Wi-Fi signals, Wi-Fi-based indoor localization techniques have emerged as a promising solution, as highlighted in various studies [17,18,19].

Wi-Fi-based indoor localization systems can generally be classified into two categories: fingerprint-based systems and parameter estimation-based systems. Fingerprint-based localization algorithms rely on extensive datasets to train predictive models. However, their accuracy is highly sensitive to environmental changes, such as human movement or fluctuations in ambient conditions like temperature and humidity. When the environment changes, these systems require data re-collection and retraining to maintain effectiveness [20,21,22,23,24,25,26]. In contrast, parameter estimation-based localization algorithms do not require training and are less affected by environmental variations. This makes them more efficient, saving significant time and effort while maintaining reliable performance in dynamic indoor environments.

Indoor localization algorithms based on parameter estimation can be further categorized into distance-based, motion velocity-based, angle of arrival (AOA)-based, and multi-parameter approaches. Various systems utilizing Wi-Fi signals have been developed to enhance localization accuracy. ArrayTrack [27] uses phase shifts between array antennas to extract AOA information, achieving high precision by increasing each access point (AP) to 16 antennas. However, this method requires expensive hardware modifications and multiple APs, limiting its practicality. SpotFi [28], on the other hand, utilizes existing Wi-Fi infrastructure without hardware modifications but still depends on multiple transceiver devices for localization. IndoTrack [29] employs commercial Wi-Fi devices and the multiple signal classification (MUSIC) algorithm to extract Doppler velocities, requiring multiple antennas for accurate results. Widar [30] estimates movement speed and direction to achieve decimeter-level accuracy but necessitates at least four transceiver devices. Similarly, Zhang et al. [31] utilized phase shifts in channel state information (CSI) to estimate Doppler shifts but required at least two sets of transceiver antennas. While these systems demonstrate promising accuracy, they often rely on specialized hardware, multiple devices, or complex setups, which can hinder scalability and ease of deployment.

**Figure 1:**
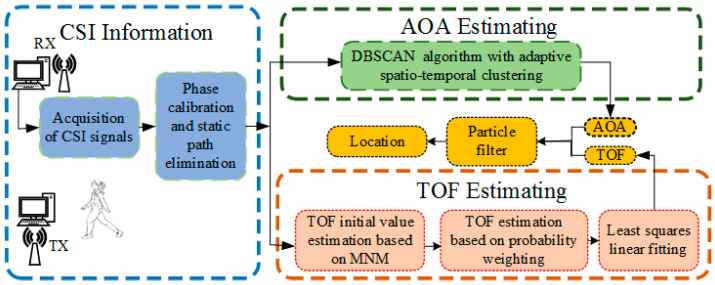
The proposed system.

Achieving precise positioning without error accumulation using a single link remains a significant challenge. This paper addresses this issue by introducing a single-link positioning system capable of decimeter-level accuracy in both positioning and tracking using only one set of transceiver antennas. The proposed system begins by processing the acquired CSI signals through phase correction and static path elimination. To estimate the initial AOA, the system employs the least mode algorithm, leveraging extended antennas for enhanced accuracy. A density-based spatial clustering of applications with noise (DBSCAN) is then applied to reduce the impact of multipath effects. For time of flight (TOF) estimation, the system initially uses the least mode algorithm to derive an initial value, followed by a weighted probabilistic TOF estimation algorithm to extract a more precise TOF for the moving target in complex multipath environments. By combining AOA and TOF data, the system estimates the target’s current position without relying on its previous position, thereby eliminating the possibility of error accumulation. The detailed system process is illustrated in Figure 1.

In summary, the main contributions of this paper compared to existing techniques are as follows:

(1) Enhanced Static Path Elimination Algorithm

This paper introduces an improved static path elimination algorithm to mitigate the impact of indoor multipath effects on parameter estimation. Existing approaches, such as those in [32], reset the antenna’s phase information to zero, discarding valuable data. In contrast, the proposed enhancement preserves critical information while effectively addressing multipath effects.

(2) Novel TOF Estimation Method

A new approach for TOF estimation of moving targets is presented, employing the (Minimum Norm Method) MNM algorithm. Additionally, a weighted probability-based TOF estimation algorithm is introduced, enabling precise extraction of flight times for moving targets in complex multipath environments.

(3) Single-Link Localization System

This study proposes a single-link Wi-Fi localization system based on CSI that eliminates error accumulation. The system’s reliability and accuracy are validated through experiments conducted in three distinct indoor environments.

The remainder of this paper is organized as follows: Section 2 provides a detailed explanation of the key algorithms proposed in this study. Section 3 discusses the results obtained from the main algorithm. Section 4 evaluates the overall system performance, and Section 5 concludes the paper with a discussion of findings and future research directions.

## 2. Materials and Methods

The proposed system, as described in the previous section, comprises several critical processes, including CSI model construction, phase calibration, static path elimination, TOF estimation using the MNM algorithm, probability-weighted TOF estimation, polynomial curve fitting, and AOA estimation and tracking. This section provides a comprehensive overview of each of these operations.

### 2.1. CSI Modeling

**Figure 2:**
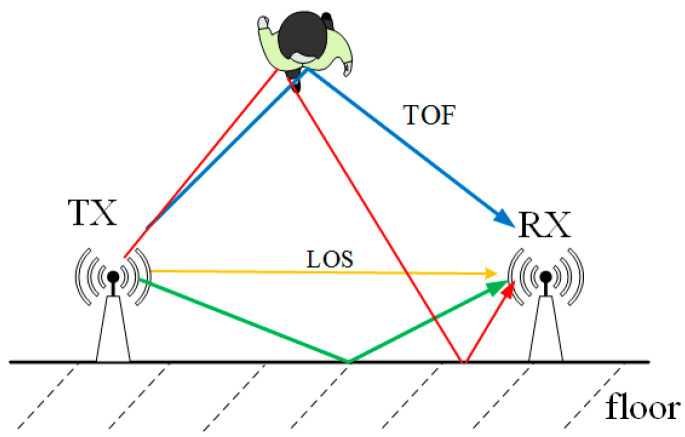
The static paths and dynamic paths.

As discussed earlier, Wi-Fi signals propagating through an indoor environment interact with objects in their path, leading to scattering. This interaction causes the CSI of the Wi-Fi signals to capture characteristics of both static and dynamic objects, along with their corresponding propagation paths. As illustrated in Figure 2, the green and yellow lines represent static paths, while the blue and red lines denote dynamic paths caused by human movement. This relationship can be mathematically expressed as follows.

This study considered a receiving array consisting of *M* elements. Let the signal received at the *m*th antenna element on the *k*th subcarrier be denoted as h(m,k,t). The received signal consists of contributions from static paths originating from stationary objects and dynamic paths resulting from a moving target. The *ls*th static path signal is denoted as $h_{ls}(m,k,t)$ and the *ld*th dynamic path signal as $h_{ld}(m,k,t)$. Mathematically, this can be expressed as

(1) $$\begin{array}{ll} h(m,k,t) & =\sum_{ls=1}^{Ls}h_{ls}(m,k,t)+\sum_{ld=1}^{Ld}h_{ld}(m,k,t)+N(t)\\ & =\sum_{ls=1}^{Ls}a_{ls}(m,k,t)e^{-j2\pi f_{i}\tau_{ls}(m,k,t)}+\\ & \sum_{ld=1}^{Ld}a_{ld}(m,k,t)e^{-j2\pi f_{i}\tau_{ld}(m,k,t)}+N(t) \end{array}$$

Let *Ls* denote the total number of static paths, and *Ld* represent the total number of dynamic paths in the environment. Here, $h_{ls}(m,k,t)$ indicates the *k*th subcarrier signal of the *m*th element from static path *ls*, while $h_{ld}(m,k,t)$ represents the *k*th subcarrier signal of the *m*th element from dynamic path *ld*. $a_{ls}(m,k,t)$ denotes the magnitude of the *k*th subcarrier signal received by the mth element from static path ls, and $\tau_{ls}$ stands for the signal flight time along static path *ls*. Similarly, $a_{ld}(m,k,t)$ denotes the magnitude of the *k*th subcarrier signal of the *m*th element from dynamic path *ld*, and $\tau_{ld}(m,k,t)$ denotes the flight time along dynamic path *ld*. Finally, *N*(*t*) represents noise in the path. Equation (1) indicates that the received signal is composed of contributions from both static and dynamic propagation paths, where static paths mainly originate from the line-of-sight component and reflections from stationary objects, while dynamic paths are primarily caused by human motion or other moving targets. These parameters collectively define the contributions of both static and dynamic paths, along with noise, to the received signal.

### 2.2. Phase Calibration and Static Path Elimination

**Figure 3:**
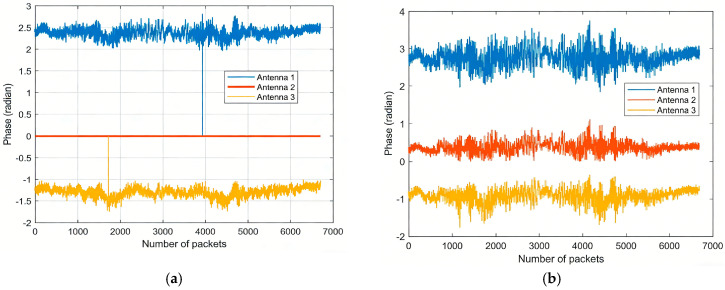
(**a**) The CSI phase uses the original static elimination algorithm. (**b**) The CSI phase uses the improved static elimination algorithm.

Due to the lack of strict clock synchronization during the reception of the CSI signal, an error arises between the measured CSI $\tilde{h}(m,k,t)$ and the actual CSI signal h(m,k,t). This discrepancy can be expressed as

(2) $$\tilde{h}(m,k,t)=h(m,k,t)e^{-2\pi j(\Delta f_{i}\varepsilon_{t}+\Delta t*\varepsilon_{f})+\zeta_{s}+n(t)}$$

where $\varepsilon_{t}$ is the time offset, $\varepsilon_{f}$ is the frequency offset, and ζ*_s_* is the initial phase offset. The time and frequency offsets are consistent across different sensors and can be eliminated using conjugate multiplication, as demonstrated in [30,32]. However, when one antenna is chosen as the reference, aligning the phases by directly multiplying all antennas by the conjugate of the reference antenna sets the reference antenna’s phase to zero, as illustrated in Figure 3a. While this method effectively removes offsets, it reduces the available phase information by one-third when calibrated data is used for motion recognition, leading to suboptimal utilization of hardware resources. In addition to the three kinds of linear noise, namely $\varepsilon_{t}$, $\varepsilon_{f}$ and ζ*_s_*, random noise *n*(*t*) also plays a significant role and cannot be ignored. The phase calibration method proposed in [30,32] wastes hardware resources and fails to effectively remove random noise. To address these limitations, this paper introduces an improved algorithm based on the methods in [30,32]. The enhanced approach is illustrated in Figure 3b, and the detailed process is as follows.

The reference signal is defined as

(3) $$h(m,k_{0},t)=\frac{1}{K}\sum_{k=1}^{K}h(m,k,t)$$

Here, *k* denotes the index of the *k*th antenna, and *K* represents the total number of antennas. Using Equation (3), part of the random noise can be eliminated. Following this, the signal contribution from the static path is removed using conjugate multiplication, expressed as

(4) $$\begin{array}{l} T(m,k,t)=\exp(j\cdot \varphi (\tilde{h}(m,k,t)))*{\tilde{h}}^{*}(m,k_{0},t)\\ =\exp(j\cdot \varphi (h(m,k,t)))*h^{*}(m,k_{0},t) \end{array}$$

where $\exp(j\cdot \varphi (\tilde{h}(m,k,t)))$ is the phase of $\tilde{h}(m,k,t)$, *k*_0_ is the reference antenna and *k* represents all antennas. The terms h(m,k,t) and $h(m,k_{0},t)$ are expressed as

(5) $$h(m,k,t)=\sum_{ls=1}^{Ls}h_{ls}(m,k,t)+\sum_{ld=1}^{Ld}h_{ld}(m,k,t)$$

(6) $$h(m,k_{0},t)=\sum_{ls=1}^{Ls}h_{ls}(m,k_{0},t)+\sum_{ld=1}^{Ld}h_{ld}(m,k_{0},t)$$

Substituting Equations (5) and (6) into Equation (4) yields

(7) $$\begin{array}{l} T(m,k,t)=\sum_{ls=1}^{Ls}\left(\exp(j\cdot \varphi (h_{ls}(m,k,t\right)))\cdot {h_{ls}}^{*}(m,k_{0},t))+\\ \sum_{ls=1}^{Ls}\left(\exp(j\cdot \varphi (h_{ls}(m,k,t\right))))\cdot \sum_{ld=1}^{Ld}({h_{ld}}^{*}(m,k_{0},t))+\\ \sum_{ld=1}^{Ld}\left(\exp(j\cdot \varphi (h_{ld}(m,k,t\right))))\cdot \sum_{ls=1}^{Ls}({h_{ls}}^{*}(m,k_{0},t))\\ +\sum_{ld=1}^{Ld}\left(\exp(j\cdot \varphi (h_{ld}(m,k,t)))\cdot {h_{ld}}^{*}(m,k_{0},t\right)) \end{array}$$

Accordingly, the phase shift caused by the static path in the system $\sum_{ls=1}^{Ls}\left(\exp(j\cdot \varphi (h_{ls}(m,k,t\right)))\cdot {h_{ls}}^{*}(m,k_{0},t))$ is of low frequency. Meanwhile, the phase shift caused by the dynamic path $\sum_{ld=1}^{Ld}\left(\exp(j\cdot \varphi (h_{ld}(m,k,t))\right)\cdot {h_{ld}}^{*}(m,k_{0},t))$ is of high frequency. These components can be effectively removed using a bandpass filter. The remaining terms can then be expanded as follows:

(8) $$\exp(j\cdot \varphi (h_{ls}(m,k,t))\cdot {h_{ld}}^{*}(m,k_{0},t)={a_{ld}}^{*}\exp(-j2\pi \Delta f_{i}(\tau_{ls}-\tau_{ld}))$$

(9) $$\exp(j\cdot \varphi (h_{ld}(m,k,t))\cdot {h_{ls}}^{*}(m,k_{0},t)={a_{ls}}^{*}\exp(-j2\pi \Delta f_{i}(\tau_{ld}-\tau_{ls}))$$

By comparing Equations (8) and (9), the following relationship can be derived:

(10) $$\begin{array}{l} \left|\exp(j\cdot \varphi (h_{ld}(k))\cdot {h_{ls}}^{*}(k_{0})\right|=\left|a_{ls}\right|\gg \\ \left|a_{ld}\right|=\left|\exp(j\cdot \varphi (h_{ls}(k))\cdot {h_{ld}}^{*}(k_{0})\right| \end{array}$$

Because static paths exhibit stable propagation geometry, lower attenuation, and more concentrated energy, their signal amplitudes are typically significantly larger than those of dynamic paths affected by blockage, scattering, and time-varying fading. Therefore, Equation (10) models the static path as the dominant component and the dynamic path as a weaker term. Since the amplitude of the static path signal $\left|a_{ls}\right|$ is significantly greater than that of the dynamic path signal $\left|a_{ld}\right|$, the latter can be neglected during processing. After eliminating the static path, the remaining signal primarily consists of minimal noise and the dynamic path signal, which significantly facilitates the extraction of accurate TOF and AOA information.

With more precise phase information and a cleaner dynamic path signal, the estimation of TOF using the MNM algorithm can proceed as follows.

### 2.3. Estimation of TOF Based on MNM Algorithm

This paper proposes a novel approach for estimating the TOF of moving targets using the MNM algorithm. The detailed algorithm process is as follows.

**Figure 4:**
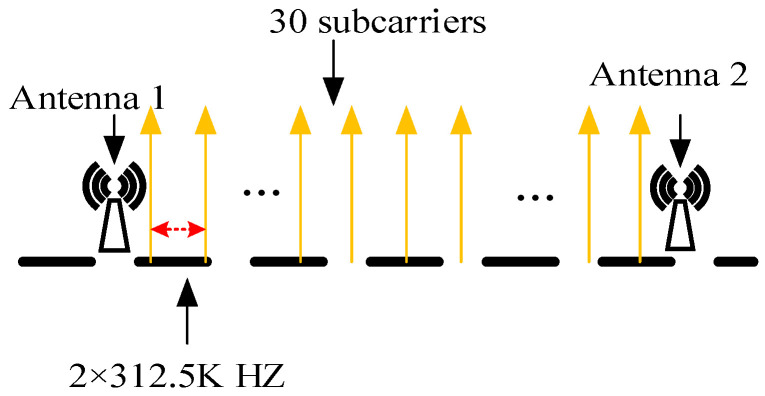
The subcarrier interval between the two antennas.

In a typical indoor environment with 6–8 multipath signals [32], each antenna operates with 30 subcarriers, providing sufficient phase deviation across subcarriers for accurate TOF estimation. In this experiment, the configuration includes channel number 165, a center frequency of 5.825 GHz, a bandwidth of 20 MHz, and subcarriers spaced at intervals of 2 × 312.5 kHz. At a distance of 3 m between the target and the transmitting and receiving antennas, the phase difference between adjacent subcarriers is approximately 0.02 radians. Over 29 subcarrier intervals, this results in a total phase deviation of 0.57 radians, which is measurable. Figure 4 illustrates the schematic diagram of the subcarrier intervals between the two antennas.

The phase shift caused by the introduction of the l path in the adjacent subcarrier can be expressed as $\Phi (\tau_{ld})=2\pi f_{\delta }\tau_{ld}$, where $f_{\delta }$ is the frequency interval between two consecutive subcarriers. For equally spaced OFDM subcarriers, the phase shift introduced by the TOF $\tau_{ld}$ relative to the first subcarrier at the *n*th subcarrier is $e^{-j2\pi (n-1)\times f_{\delta }\tau_{ld}}$.

For a typical 5300 network card with three receiving antennas, the guiding vector of the signal in the path *ld* can be expressed as

(11) $$\vec{a}(\theta_{ld})={\left[\overset{antenna1}{\overset{︷}{1,\Phi (\tau_{ld}),\ldots ,\Phi {\left(\tau_{ld}\right)}^{I-1}}},\underset{antenna2}{\underset{︸}{1,\Phi (\tau_{ld}),\ldots ,\Phi {\left(\tau_{ld}\right)}^{I-1}}},\overset{antenna3}{\overset{︷}{1,\Phi (\tau_{ld}),\ldots ,\Phi {\left(\tau_{ld}\right)}^{I-1}}}\right]}^{T}$$

Here, *I* represents the total number of subcarriers of each antenna. The total guiding vector of the *L*-path can be expressed as

(12) $$\vec{a}={\left[\vec{a}(\theta_{1})\;\vec{a}(\theta_{2})\ldots \vec{a}(\theta_{L})\right]}^{T}$$

**Figure 5:**
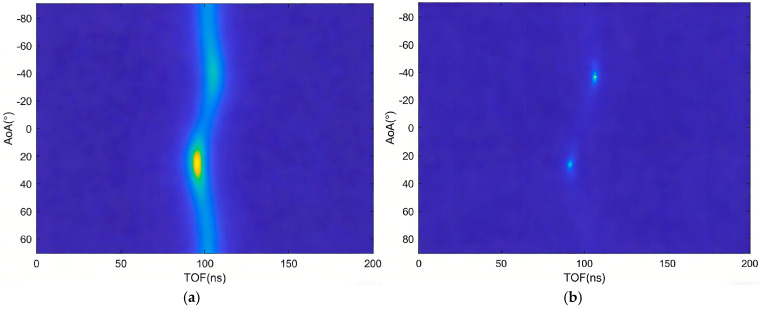
(**a**) The AOA and TOF are estimated by the MUSIC algorithm. (**b**) The AOA and TOF are estimated by the MNM algorithm.

The MNM algorithm is a subspace algorithm with constrained weights, offering higher resolution compared to the MUSIC algorithm, as illustrated in Figure 5a,b. In this study, simulated signals with angles of −30° and 30° were used to evaluate the performance of both algorithms. After identical preprocessing, the MUSIC and MNM algorithms were applied for joint AOA and TOF estimation. The simulation results demonstrate that the MNM algorithm achieves significantly higher angular resolution than the MUSIC algorithm under identical conditions, highlighting its superiority in resolving closely spaced angles.

The constraint conditions for the MNM algorithm are as follows:

(13) $$\left\{\begin{array}{c} \min\left\{W^{H}W\right\}\\ W(1)=1,U_{s}W=0 \end{array}\right.$$

Here, $U_{s}$ is constructed using the signals from the dynamic path, and *W* represents a linear combination of the noise space. The TOF is estimated by identifying the peak of the following formula:

(14) $$p_{MNM}=\frac{1}{{\left|a^{H}W_{MNM}\right|}^{2}}$$

All CSI data were divided into short time intervals with a sampling rate of 1000 per second. Each data segment is represented as a 90 × *s* matrix, where 90 represents the total number of subcarriers and *s* represents the number of snapshots in the interval. The initial TOF value is then estimated using the MNM algorithm, leveraging the phase and amplitude information within each data segment.

### 2.4. A Probability-Weighted TOF Estimation Algorithm

The directly obtained TOF value is often inaccurate due to complex multipath effects in indoor environments. To refine the TOF estimation, this method leverages the stability of reflected signals from the target, which are more concentrated compared to the dispersed multipath signals. The true TOF is identified based on the density of points in the distance space, where higher-density regions are closer to the true value. Additionally, a probability-weighting approach incorporates the TOF estimate from the previous time interval, enhancing temporal consistency. This combined approach of density analysis and probability weighting effectively suppresses noise and multipath interference, yielding more precise TOF measurements. The specific steps for refining TOF estimation are as follows:

Step 1: Removing Outliers: Filter out TOF values exceeding a predefined threshold *T*, determined by the indoor space size, as they are physically implausible.

Step 2: Interpolation Complement Points: For missing points at the beginning of the sequence, replace them with the nearest available points. For missing points in the middle, replace them with the average of the preceding and succeeding points. For missing points at the end, replace them with the nearest available points.

Step 3: Distance Matrix Calculation: Compute the time-space distance matrix between each point and all other points. Identify the *E* nearest points for each data point and calculate their sum to assess the density around each point as

(15) $$dis(q)=\sum_{i=1}^{E}neighb(d(q))$$

The distance formula can be defined as

(16) d(q)=norm(tof(q)−tof(e))∗α+norm(t(q)−t(e))∗β.

where dis(q) represents the sum of distances between the *q*th point and its nearest *E* points, and neighb() represents the nearest points. Meanwhile, d(q) represents the distance between the *q*th point and the eth point, α and β are weight parameters, and norm represents the Euclidean distance between two points. In Equations (16) and (17), the parameters α and β denote the weights of spatial and temporal distances, respectively. Their values are determined via a traversal (grid-search) experimental strategy, with the optimal performance obtained when both weights are set to 0.5, ensuring a balanced contribution of spatial and temporal information.

Step 4: Confidence Probability Calculation: The confidence probability of each observed value can be calculated according to dis(q):

(17) $$\begin{array}{l} p(\tilde{t}x_{q}=tz_{q})=\left\{\begin{array}{ccc} \;\;\;\;\;\;\;\;\;\;\;\;\;\;\;\;\;\;\;\;\;\;\;1 & if & dis(q)<Amp1\\ 1-\frac{dis(q)-\frac{1}{N}\sum_{n=1}^{N}dis(n)}{dis(q)} & if & \;\;\;\;\;\;\;\;\;\;\;\;\;\;Amp1\leq dis(q)\leq Amp2\\ \;\;\;\;\;\;\;\;\;\;\;\;\;\;\;\;\;\;\;\;\;\;\;0 & & \;\;\;\;\;\;\;\;\;\;\;\;\;\;\;\;\;\;\;\;\;\;\;\;\;\;others \end{array}\right. \end{array}$$

where $\tilde{t}x_{q}$ is the estimated TOF value of the *p*th point, $tz_{q}$ is the TOF value of the *p*th point after interpolating the complement points directly with the MNM algorithm, and *N* is the point with a probability of 1. To determine the values of Amp1 and Amp2, the probability density function f(x) of dis(q) was first estimated using the best-fit algorithm. If f(x) conforms to a normal distribution, its probability density function can be expressed as

(18) $$f(d)=\frac{1}{\sigma \sqrt{2\pi }}\exp(\frac{{\left(d-\mu \right)}^{2}}{2\sigma^{2}})$$

Then, Amp1 and Amp2 can be determined using the following formulas:

(19) $$\int_{-\infty }^{Amp1}f(x)dx=0.2$$

(20) $$\int_{-\infty }^{Amp2}f(x)dx=0.9$$

Although *f*(*d*) is assumed to follow a normal distribution, the cumulative density values of 0.2 and 0.9 are chosen to suppress low-amplitude noise while retaining most informative signal components and excluding outliers.

Step 5: TOF Value estimation: To estimate the TOF values before and after the point with an observation probability of 1, use the following formulas:

(21) $$tx_{q}=tx_{q-1}\pm \frac{v_{mean}*\Delta t}{c}$$

(22) $$\tilde{t}x_{q}=(1-p(\tilde{t}x_{q}=tz_{q}))*tx_{q}+p(\tilde{t}x_{q}=tz_{q})*tz_{q}$$

where $v_{mean}$ represents the normal speed of human movement, Δt represents the time interval of adjacent data, and *c* is the speed of light. These formulas calculate the weighted average of TOF values, using the confidence probabilities in (18) as weights, for points occurring before and after the high-confidence point. This ensures continuity and robustness in the TOF estimation.

Step 6: Optimize the Estimated Value: The curve fitting algorithm refines TOF estimates by leveraging the continuity of human movement. Fitting a smooth curve, such as a polynomial or spline, to the estimated values helps reduce noise and improve accuracy. This ensures that the estimated values remain consistent with realistic motion trajectories, as the direction of human movement is typically continuous.

(23) $$Y(r)=a_{0}+a_{1}r+a_{2}r^{2}+\cdots +a_{n}r^{n}$$

(24) $$\sum_{q=1}^{Q}{\left(f(t_{q})-TOF_{q}\right)}^{2}=\min$$

Y(x) is the target polynomial used for curve fitting, $t_{q}$ is the TOF value before fitting and $TOF_{q}$ is the TOF value after curve fitting, and *Q* is the number of the total points. This process smooths and refines TOF estimates.

**Figure 6:**
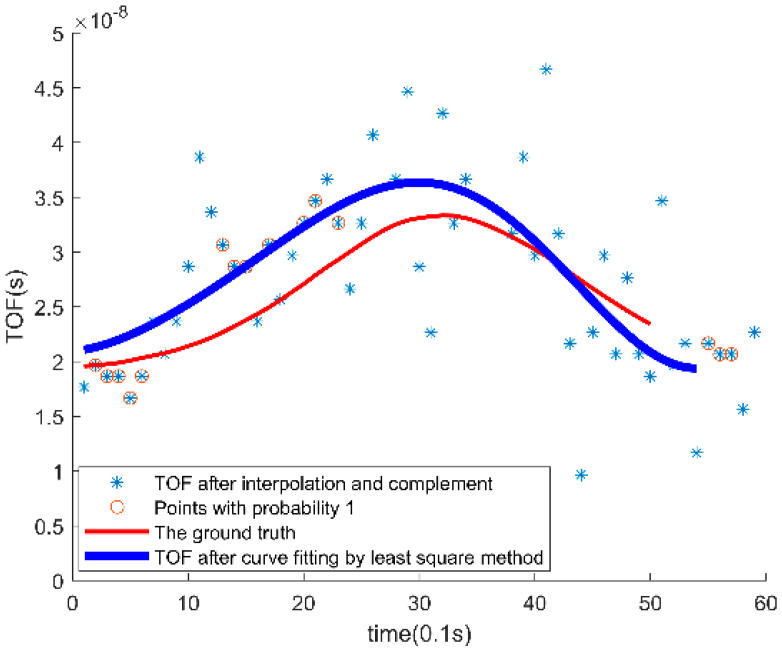
Data processing results.

Figure 6 presents the results of the probability-weighted TOF estimation algorithm. The blue asterisks represent TOF values after point complementation, which show significant fluctuations. The red circles indicate points with an observation probability of 1, where higher density indicates closer proximity to the true value. The red lines represent the actual TOF values, serving as the reference, while the blue lines illustrate the results of least square curve fitting after applying probability weighting, providing a smoother and more accurate TOF estimation.

### 2.5. Adaptive Spatio-Temporal Clustering for AOA Estimation

Previous studies have primarily focused on AOA estimation within a spatial context over short durations, often neglecting the temporal continuity of AOA. To address this gap, this paper employs an adaptive parameter-tuned DBSCAN spatio-temporal clustering algorithm, which incorporates both spatial and temporal continuity of AOA. The detailed process involves three steps [33]:

Step 1: Initial AOA information acquisition: The initial AOA values are obtained using a joint estimation algorithm that combines both AOA and TOF.

Step 2: DBSCAN spatio-temporal clustering: The initial AOA values often contain substantial multipath and noise data. To address this, the paper employs an adaptive parameter-tuning DBSCAN spatio-temporal clustering algorithm. Specifically, within a temporal window of *t* ms, 20 instantaneous AOA estimates are generated per millisecond, forming *t* × 20 angle samples, which are then clustered using DBSCAN to obtain a robust AOA estimate by selecting the centroid of the densest cluster. A comprehensive description of the input representation, adaptive selection of eps and MinPts, and the adopted distance metric can be found in Ref. [33]. In the DBSCAN-based AOA enhancement, core points and their associated border points within the dominant cluster are retained to compute the cluster centroid as the enhanced AOA estimate, while noise points are discarded. This method filters the data to yield more accurate AOA estimates.

Step 3: Post-clustering data processing: During the initial phase of movement, the dynamic path estimated by the aforementioned algorithm may be inaccurate due to the low speed, which can cause the angle of the dynamic path to be unstable or even undetectable. Therefore, additional processing of the AOA data is necessary. This includes interpolating complementary points and applying linear fitting techniques to enhance accuracy and stability.

### 2.6. Trajectory Tracking

**Figure 7:**
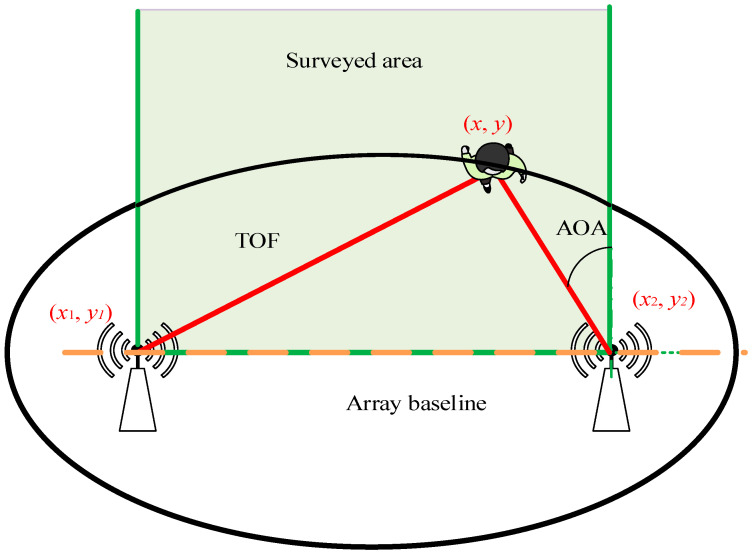
The location and tracking diagram.

After obtaining the values of TOF and AOA, the position of the moving target can be uniquely determined within the detection area, as illustrated in Figure 7. The red line represents the TOF of the target, while the purple line depicts the AOA relative to the green coordinate system. By combining AOA and TOF, the target’s position can be precisely located within the detection area, which is defined by an ellipse representing the search area. If the target position is (x,y), the positions of the transmitting and receiving antennas are $\left(x_{1},y_{1}\right)$ and $\left(x_{2},y_{2}\right)$, respectively. The following equation can be used to calculate the target coordinates:

(25) $$\left\{\begin{array}{c} \tan\theta_{1}=\frac{y-y_{2}}{x-x_{2}}\\ \frac{x^{2}}{{\left(F_{1}F_{2}/2\right)}^{2}}+\frac{y^{2}}{{\left(c\cdot t\right)}^{2}-{\left(F_{1}F_{2}/2\right)}^{2}}=1 \end{array}\right.$$

where $F_{1}F_{2}$ represents the spacing between the transceiver antennas and *t* is the measured flight time.

### 2.7. The Algorithm’s Complexity

For time-of-flight (TOF) estimation, this paper employs a processing unit of 0.1 s data (90 × 100 dimensions, where 90 represents the number of subcarriers across three antennas and 100 denotes the 100 data packets contained within 0.1 s). For *M* = 100 (100 snapshots) and *N* = 90 (90 array elements), the covariance matrix has dimensions of *M* × *N* × *N*, with a computational complexity of *O* (*M* × *N*^2^). The eigen decomposition of the 90 × 90 covariance matrix incurs a complexity of *O* (*N*^3^). Constructing the minimum-norm vector in the noise subspace typically requires *O* (*N*^3^) operations. For spectral peak search, assuming *K* TOF points are evaluated (set to 100 in this work), the complexity per point is *O* (*N*), yielding a total complexity of *O* (*K* × *N*). Distance computation involves *T* time steps (ranging from approximately 50 to 120 in this study), with traversal complexity of *O* (*T*^2^). The computational costs of other steps are negligible. The overall algorithm complexity is formulated as *O* (*N*^3^ + *M* ×*N*^2^ + *K* × *N* + *T*^2^). Substituting the parameters (*N* = 90, *M* = 100, *K*= 100, *T* ≈ 50–120), the estimated total computational complexity is approximately 1.6 × 10^6^ operations.

Angle of Arrival (AOA) estimation employs a joint estimation of AOA and TOF, with algorithmic complexity analogous to that of TOF estimation. The complexity is approximately *O* (*N*^3^ + *M* × *N*^2^ + *K* × *N* × *L*), where *L* denotes the AOA search range (typically 180 points in this study). The clustering process in this study operates on a 1 ms time window, with 40 estimated AOA values extracted per window. For 1 s of data, this results in 4 × 10^4^ points requiring clustering. The computational complexity of the DBSCAN clustering algorithm is approximately *O* (*N*^2^), necessitating 1.6 × 10^9^ distance calculations and neighborhood search operations. Assuming a standard CPU (e.g., Intel Core i7-12700K, 3.6 GHz, single-core performance ~10^10^ FLOPS), the estimated processing time is 1.6 s, which challenges real-time processing feasibility. To address this, algorithmic optimizations such as spatial indexing structures (e.g., KDTree) can reduce complexity to *O* (*N* log *N*), achieving a processing time of 0.4 ms, thereby satisfying real-time requirements.

## 3. Results

The proposed algorithm was validated and tested through three experiments using data from Tsinghua Widar2.0. Representative photographs of the test environments, spatial distributions of the collected data points, and schematic illustrations of typical movement trajectories can be found in Ref. [32]. The experimental system is implemented based on Widar2.0 using a pair of off-the-shelf laptops equipped with Intel 5300 NICs. The transmitter employs a single antenna to broadcast packets, while the receiver is equipped with three antennas forming a uniform linear array. CSI measurements are collected using the Linux 802.11n CSI Tool with devices operating in monitor mode on channel 165 (5.825 GHz) and a packet transmission rate of 1000 Hz. The dataset comprised 24 trajectories, each providing approximately 70 angle and flight time measurements, resulting in about 1700 data points. The 24 trajectories included 6 “Z”-shaped paths facing three directions, 7 circular paths starting from four locations, 2 symmetrical “7”-shaped paths, 1 rectangular path, 6 vertical lines from two starting points, and 2 diagonal lines from two starting points. This diverse setup effectively assesses the algorithm’s efficiency and accuracy across various real-world environments.

The proposed algorithm demonstrates robust performance on commercially available Wi-Fi devices and operates without requiring specialized equipment for CSI acquisition. Specifically, it is compatible with standard Wi-Fi network interface cards (NICs), including widely deployed models such as the Intel 5300 and Atheros AR9580, enabling CSI extraction directly from existing infrastructure without additional hardware modifications. Through optimized CSI processing pipelines incorporating noise suppression and feature enhancement techniques, the algorithm achieves sub-meter localization accuracy (error < 1 m) in typical indoor environments, satisfying practical application requirements. Computational efficiency is ensured via a lightweight architecture that adheres to the processing capabilities of commercial off the shelf (COTS) devices, eliminating dependencies on dedicated accelerators or supplementary computing resources. By leveraging ubiquitous Wi-Fi infrastructure, the solution offers low-cost deployment and seamless scalability to large-scale scenarios. These attributes collectively underscore the algorithm’s practical viability and broad applicability in real-world implementations.

### 3.1. Accuracy of TOF Estimation

This paper first evaluates the execution time of the proposed algorithm across various packet lengths, comparing it with that of the Widar2.0 algorithm, as shown in Table 1. When the packet count is 2000, the execution time of the proposed algorithm is approximately half that of the Widar2.0 algorithm. However, as the packet count increases to 14,000, the execution time of the proposed algorithm is reduced to roughly one-seventh of that of Widar2.0. This significant improvement in efficiency stems from the proposed algorithm’s reliance on a one-dimensional search, which is computationally more efficient compared to the multi-dimensional search used in Widar2.0.

**Figure 8:**
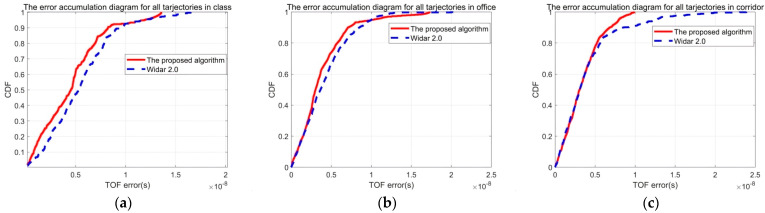
The cumulative distribution function (CDF) plots of TOF estimation errors. (**a**) The CDF in the classroom. (**b**) The CDF in the office. (**c**) The CDF in the corridor.

To demonstrate the universal applicability of the algorithm proposed in this paper, experiments were conducted on all data collected across three distinct environments using the Widar2.0 framework. Figure 8a–c present the cumulative distribution function (CDF) plots of TOF estimation errors for the classroom, office, and corridor environments, respectively. The experimental results presented in Figure 8 demonstrate significant variations TOF detection accuracy across different environments. Specifically, the classroom environment exhibited the highest median TOF detection error of 4.8 ns (nanoseconds), followed by the office setting with a median error of 3.7 ns. The most accurate measurements were achieved in the hallway configuration, where the median error decreased to 3.1 ns. This comparative analysis reveals a clear environmental dependency in TOF measurement precision. The experimental data reveal that the classroom environment exhibits the largest ranging error (5.6 m transmitter–receiver separation), with this degraded performance attributed to extended antenna spacing exacerbating multipath propagation effects. Specifically, the 148.9% greater inter-antenna distance compared to other test configurations (average 2.25 m) induces greater multipath component delays and spatial signal decorrelation. This phenomenon demonstrates strong correlation with the theoretical framework established in Section 4.1 (“Environmental Impacts on Tracking Fidelity”). These plots highlight the algorithm’s performance in diverse settings, emphasizing its adaptability and accuracy across varying conditions.

These improvements in accuracy can be attributed to the use of 90 array signal antennas in the proposed algorithm, which is three times the number of antennas employed by the algorithms referenced in [32] for TOF estimation. Additionally, the integration of probability weighting and linear fitting algorithms further enhances the accuracy of TOF estimates by effectively refining the results, ensuring they align more closely with the true values.

### 3.2. Accuracy of Positioning Accuracy

**Figure 9:**
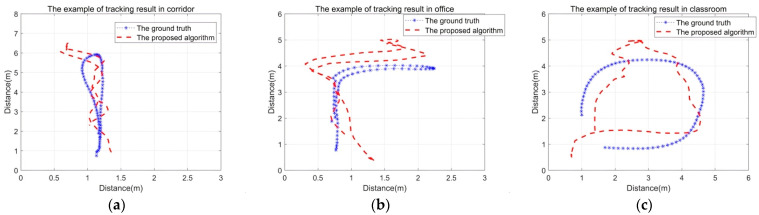
The tracking results for different shapes of trajectory. (**a**) The line trajectory in the corridor. (**b**) The ‘7’shaped trajectory in the office. (**c**) The round-shaped trajectory in the classroom.

The proposed algorithm evaluates all trajectory localization errors across three distinct environments within the Widar2.0 framework. Representative examples of the localization outcomes for these environments are presented in Figure 9. Specifically, Figure 9a illustrates the classroom environment, Figure 9b depicts the office environment, and Figure 9c portrays the corridor environment. As demonstrated in Figure 8, the proposed algorithm achieves enhanced localization accuracy across diverse environments, exhibiting median errors of 0.45 m, 0.5 m, and 0.6 m in corridor, office, and classroom environments, respectively. The observed error magnitudes correlate with antenna spacing, detection range, and environmental complexity, consistent with prior analytical predictions.

### 3.3. Comparisons with Other Localization Algorithms

**Figure 10:**
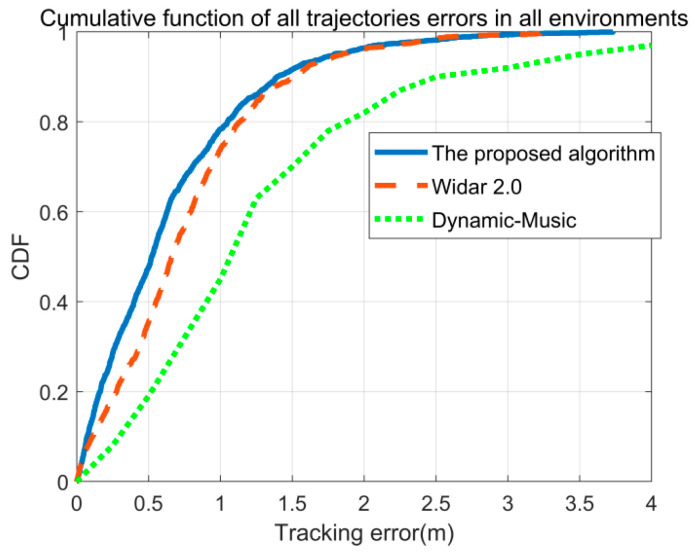
The CDF of tracking errors with different algorithms.

Using the same dataset, this study compares localization errors across various algorithms in an integrated setting. Figure 10 illustrates the localization errors of the proposed algorithm, Widar2.0, and Dynamic-Music across three environments. The mean localization error of the proposed algorithm is 0.63 m, outperforming Widar2.0 at 0.75 m and Dynamic-Music at 1.1 m. The superior performance of the proposed algorithm stems from its comprehensive consideration of the temporal correlation between AOAs and TOFs before and after events. This approach enables more accurate localization and trajectory tracking, particularly in complex indoor environments.

## 4. Discussion

To gain a deeper understanding of the system performance of the proposed algorithm, analyses were conducted focusing on three key influencing factors: environmental conditions, data sampling rates, and trajectory shapes. These evaluations aim to assess how variations in these factors affect the accuracy and efficiency of the system, providing insights into its robustness and adaptability across diverse scenarios.

### 4.1. The Influence of Environments on Tracking Accuracy

**Figure 11:**
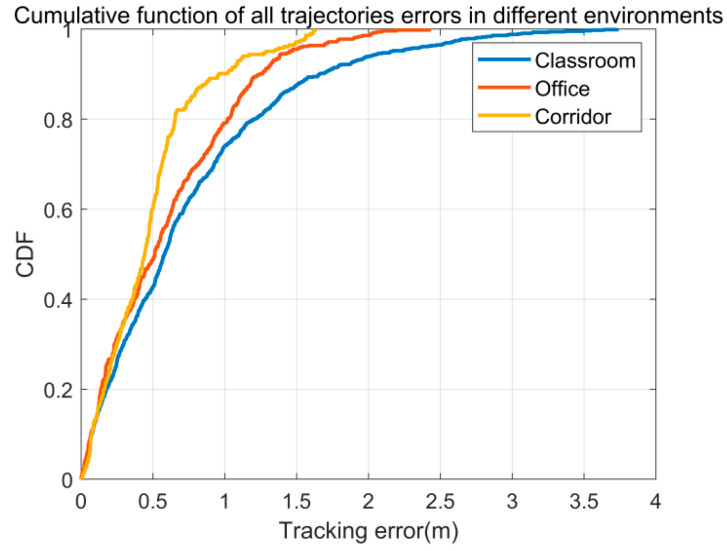
The CDF of tracking errors versus different environments.

This experiment analyzed the localization errors across the three test environments in Widar2.0. Figure 11 presents the cumulative distribution function (CDF) of error accumulations for these environments. The classroom environment exhibits the largest error, primarily due to increased multipath interference and the wider spacing of antennas, which allows the detection of weaker Doppler shifts but introduces additional challenges. Moreover, the error is influenced by the size of the detection area: the classroom has a detection area of 30 square meters, the hallway is 20 square meters, and the office is 10 square meters. Larger detection areas tend to result in greater errors, highlighting the impact of environmental factors on system performance.

### 4.2. The Influence of Sampling Rates on Tracking Accuracy

**Figure 12:**
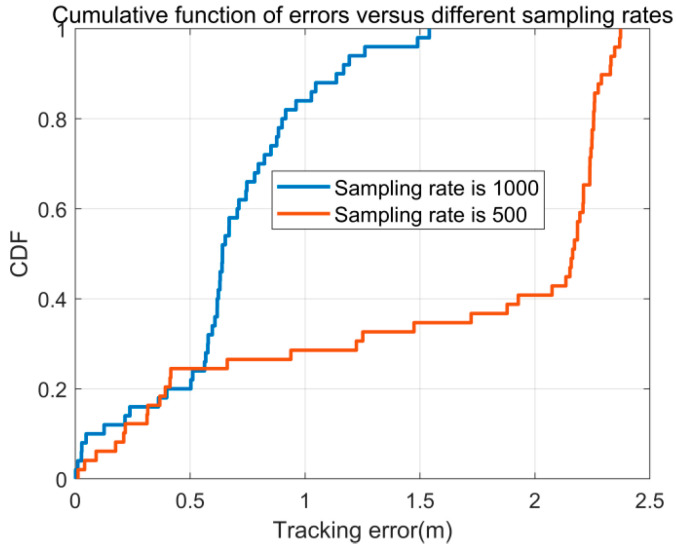
The CDF of tracking errors in different sampling rates.

In the context of the proposed algorithm, packets are collected at a rate of 1000 packets per second (pps). A relevant question arises: Can accurate localization still be achieved at lower sampling rates? To explore this, the study compared localization errors at sampling rates of 500 pps and 1000 pps, as shown in Figure 12. The results reveal a significant increase in localization error when the sampling rate is reduced, indicating that the algorithm’s accuracy decreases at lower sampling rates. This suggests that maintaining a higher sampling rate is critical for achieving precise localization.

### 4.3. The Influence of the Shapes of Trajectory on Tracking Accuracy

**Figure 13:**
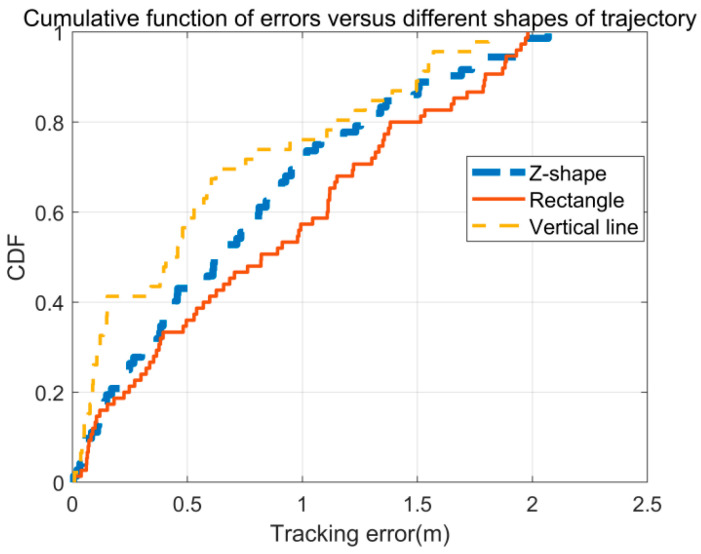
The CDF of tracking errors in different shapes of trajectory.

Do variations in trajectories affect position estimation? To address this question, the study compared position errors across three distinct trajectories: a ‘Z’-shaped trajectory, a rectangular trajectory, and a straight-line trajectory. As depicted in Figure 13, the straight-line trajectory exhibits the smallest error attributed to the continuous and consistent position information obtained during linear motion, which enhances accuracy. In contrast, the ‘Z’-shaped trajectory, with two turns, introduces significant changes in information at each turn, leading to larger errors. The rectangular trajectory, involving three turns, results in the highest localization error due to the frequent disruptions in motion continuity. These findings highlight the influence of trajectory complexity on positioning accuracy.

Table 2 compares four mainstream indoor positioning technologies. The proposed Wi-Fi CSI-based method achieves a localization accuracy of 0.63 m using existing Wi-Fi access points, offering a favorable balance between accuracy, cost, and device compatibility. This makes it well suited for large-scale indoor environments with pre-deployed Wi-Fi infrastructure, such as offices, shopping malls, and residential buildings. In contrast, UWB provides higher accuracy at the expense of dedicated hardware and higher cost, while Bluetooth and RFID suffer from either dense infrastructure requirements or limited real-time performance. Overall, the proposed method demonstrates strong practicality for large-scale civilian indoor localization applications.

## 5. Conclusions

This paper introduces a novel, real-time indoor localization framework that leverages single-link Wi-Fi signals to achieve decimeter-level accuracy in complex and dynamic indoor environments. Addressing key challenges such as multipath interference and error accumulation found in conventional methods, the system employs a series of advanced signal processing and AI-enabled techniques. These include refined phase calibration, static path suppression, and an adaptive, unsupervised clustering algorithm for spatio-temporal feature extraction. To enhance localization precision, the system integrates a minimum norm-based angle-of-arrival (AOA) estimator with a probability-weighted time-of-flight (TOF) refinement algorithm, enabling robust positioning without dependence on historical trajectory data or offline training. Experimental evaluations conducted across three representative environments—classroom, office, and corridor—demonstrate consistent performance gains over state-of-the-art baselines such as Widar2.0 and Dynamic-MUSIC. The proposed approach achieves an average localization error of 0.63 m, significantly outperforming Widar2.0 (0.75 m) and Dynamic-MUSIC (1.1 m). The framework exhibits strong adaptability to variations in sampling rates, path geometry, and environmental complexity, highlighting its potential for real-world deployment. Unlike traditional deterministic models, the proposed architecture incorporates intelligent, self-adjusting mechanisms based on AI principles such as unsupervised learning and probabilistic inference, allowing dynamic adaptation to environmental uncertainty without the need for retraining.

Looking ahead, future work will explore multi-target localization in overlapping signal scenarios, integration with vertical antenna arrays for full 3D positioning, and sensor fusion with inertial and visual data to further enhance robustness in occluded or cluttered environments. These extensions aim to broaden the framework’s applicability to next-generation AI-driven applications such as autonomous indoor logistics, human–robot interaction, and smart infrastructure management.

## Figures and Tables

**Table 1:** A comparison of running time for different algorithms.

Number of Packets	Widar2.0	Improved Algorithms
2000	1396 ms	733 ms
5000	3900 ms	1036 ms
10,000	8733 ms	1474 ms
14,000	13,510 ms	1821 ms

**Table 2:** Key metrics comparison of indoor positioning technologies.

Technology	Accuracy	Infrastructure Needs	Cost	Device Compatibility
proposed	0.63 m	Wi-Fi APs	low	high (Wi-Fi devices)
UWB	0.1 m	dedicated anchors	high	low
Bluetooth	1–3 m	beacons	medium	high (BLE support)
RFID	1–5 m	Readers/Tags	medium	low (tag-based)

## Data Availability

The original contributions presented in this study are included in the article. Further inquiries can be directed to the corresponding author.
